# Chloride of Zinc Moulded into Sticks for the Purpose of Cauterization

**Published:** 1861-01

**Authors:** 


Chloride of Zinc Moulded into Sticks for the Purpose of Cauterization.—Soften gutta-percha in boiling alcohol, and incorporate it with finely-pulverized chloride of lime, in a warm porcelain mortar, taking equal parts of each. Then roll rapidly on a porphyry slab, to the diameter of a quill, and divide in fragments, each of which shall be pointed at one end. Keep these in a wide-mouthed bottle in powdered lime. These sticks remain perfectly hard, are easily handled, cauterize with great regularity, and act as a sponge through which the chloride will slowly exude, becoming liquid by the action of the air and the skin.—*Lancet.*

